# Efficacy of vancomycin in combination with various antimicrobial agents against clinical methicillin resistant *Staphylococcus aureus* strains

**DOI:** 10.12669/pjms.37.1.2887

**Published:** 2021

**Authors:** Gulseren Aktas

**Affiliations:** 1 Dr. Gulseren Aktas, Ph.D. Associate Professor, Department of Medical Microbiology, Istanbul University, Istanbul Faculty of Medicine, 34093, Capa-Istanbul, Turkey

**Keywords:** Conventional antibiotics, new generation antibiotics, combination, E-test, MRSA

## Abstract

**Background:**

Multi-drug resistant methicillin resistant Staphylococcus aureus (MRSA) strains that have been isolated frequently worldwide have difficulties in the treatment and therefore alternative choices for the treatment of the infections are required. The aim of the study was to evaluate the interaction of various antimicrobials in combination with vancomycin against MRSA.

**Methods:**

Twenty five clinical MRSA strains isolated in 2016 were included in the study. The interaction between vancomycin and new generation/conventional antimicrobials against MRSA strains was analyzed by E-test.

**Results:**

All of the strains tested was found to be susceptible to vancomycin, telavancin, dalbavancin, ceptobiprole, daptomycin, linezolid, quinupristin-dalfopristin, trimethoprim-sulfamethoxazole, rifampicin and tigecycline. The susceptibility rates of the isolates were found to be high, with the lowest rate (48%) against azithromycin. According to the fractional inhibitory concentration index results, synergistic interaction with vancomycin was determined with trimethoprim-sulfamethoxazole, azithromycin, linezolid, minocycline, dalbavancin, clindamycin in five, three, two, two, one, one and one strain(s), respectively. Additionally, all combinations studied showed additive interaction at high rates.

**Conclusions:**

The results of the study indicate that the use of vancomycin in combination with conventional and new generation antibiotics is promising.

## INTRODUCTION

Multiple antibiotic resistant Gram-positive pathogens have been causing severe problems in the treatment since 1980s.^1^,^2^ Methicillin-resistant *Staphylococcus aureus* (MRSA) remains an important cause of serious infections, particularly among hospitalized patients, and it has recently been observed in an increasing trend in community settings.^3^,^4^ Vancomycin is often recommended as the first-choice antibiotic in the treatment of MRSA infections. However, emergence of resistance during therapy has been recognized.^5^,^6^ Although vancomycin remains the preferred agent for the treatment of MRSA infections, newer antimicrobials seem to be attractive for therapeutic options.^7^,^8^ On the other hand, although the newer antibiotics such as dalbavancin, telavancin, daptomycin, linezolid, tigecyclin and quinupristin/dalfopristin have been successfully used in the treatment of the infections, the presence of resistance to these antibiotics was also reported.^9^ The use of the combinations of antibiotics is known to increase the success of treatment with the synergistic interaction. Moreover, combination of antibiotics in the treatment has the advantage of reducing or delaying the emergence of resistant strains and reducing the toxicity.^10^ The aim of the present study was to evaluate the synergistic potential of vancomycin in combination with both conventional and new generation antimicrobials against MRSA strains that would give an idea for the treatment of MRSA infections.

## METHODS

Twenty five MRSA strains which were randomly selected were included in the study. Each strain was isolated from different patients who were admitted to the different wards of the Istanbul University, Istanbul Faculty of Medicine’s Hospital, through 2016. A total of 25 randomly-selected clinical MRSA strains were included in the study. Strains were isolated from generally blood, pass, sputum and lesser urine samples of the patients. The identification of the strains and the determination of methicillin resistance were performed using conventional methods.^11^,^12^ Once an organism had been characterized as a Gram positive cocci with a Gram-stained preparation, it was further identified in a series of tests, which were involved catalase, plasma coagulase, DNase, and resistance to cefoxitin (30 mg; Oxoid, England) by using the disk diffusion method. Strains that were positive in the tests mentioned above were identified as methicillin-resistant *Staphylococcus aureus* (MRSA). Strains were stored frozen at -70°C in Brain Heart Infusion broth (Oxoid, England) with 20% glycerol before testing. The antibiotics used in the study were listed in the Table-I.

**Table-I:** Conventional and newer antimicrobial agents used in the study.

Conventional Antimicrobial Agents	Newer Antimicrobial Agents

Vancomycin (VA)	Telavancin (TLV)
Amikacin (AK)	Dalbavancin (DAL)
Tobramycin (TOB)	Daptomycin (DAP)
Azithromycin (AZM)	Linezolid (LNZ)
Clindamycin (CD)	Ceftaroline (CPT)
Minocycline (MN)	Ceftobiprole (BPR)
Trimethoprim-sulfamethoxazole(SXT)	Quinupristin-Dalfopristin (QDA)
Rifampicin (RIF)	Tigecycline (TGC)

The minimum inhibitory concentrations (MICs) of the conventional antibiotics such as vancomycin (VA), amikacin (AK), tobramycin (TOB), azithromycin (AZM), clindamycin (CD), minocycline (MN), trimethoprim-sulfamethoxazole (SXT), rifampicin (RIF), and the MICs of new generation such as telavancin (TLV), dalbavancin (DAL), daptomycin (DAP), linezolid (LNZ), ceftaroline (CPT), ceftobiprole (BPR), quinupristin-dalfopristin (QDA), and tigecycline (TGC) against 25 clinical MRSA strains were determined using E-tests in accordance with the recommendations of the manufacturing company (LIOFİLCHEM^®^ s.r.l., Italy).

### Identification of MIC values

Mueller-Hinton II Agar (MHA) (BBL™, USA) was used for determining the MICs. The strains that were incubated overnight were adjusted to the turbidity of 0.5 McFarland in Mueller Hinton broth using densitometry (Biosan, Riga, Latvia) and 200 µL of the suspension was spread onto the MHA agar plate. After drying, E-test strips were placed, and the media were incubated for 24 h at 35°C.^10^ The MIC was interpreted as the value at which the inhibition zone intersected the scale on the E-strip. The determination of the susceptibilities of the strains against antimicrobials that were tested was performed according to the MIC breakpoints recommended by European Committee on Antimicrobial Susceptibility Testing (EUCAST).^12^ *Staphylococcus aureus* ATCC 29213, and *Enterococcus faecalis* ATCC 29212 were used for the quality control in all experiments.^12^,^13^

### Antibiotic combination interactions

The combination of each antimicrobial agent with vancomycin was evaluated by the E-test. The preparation of the inoculum and the streaking of Mueller-Hinton agar plates were as previously described. The E-test strips were placed on the Mueller-Hinton agar in a cross formation with a 90° angle at the intersection between the scales at their respective MICs.^14^ The plates were then incubated for 24 hour at 35°C. After incubation, the zones of inhibition were read for each E-test seperately at the intersection of the zone with the E-strip as described above for the determination of the MIC results. And then, the fractional inhibitory concentration (FIC) index (FICI) values were calculated by using the following formula:

A/MIC_A_+B/MIC_B_=FIC_A_+FIC_B_=FICI

### In this formula;

A: identifies the MIC value of antibiotic A when tested in combination with antibiotic B,

MIC_A_: identifies the MIC value of antibiotic A alone,

FIC_A_: identifies the fractional inhibitory concentration of the antibiotic A.

B: identifies the MIC value of antibiotic B when tested in combination with antibiotic A,

MIC_B_: identifies the MIC value of antibiotic B alone,

FIC_B_: identifies the fractional inhibitory concentration of the antibiotic B.

The results of FICI ≤ 0.5 were evaluated as synergistic, FICI > 0.5 - < 2 were evaluated as additive, FICI ≥ 2 - < 4 as indifference, and FICI ≥ 4 as antagonistic interaction.^15^

### Ethics Approval

This being a retrospective study did not need ethics approval.

## RESULTS

The MIC_50_, MIC_90,_ MIC_interval_ values and susceptibility rates are given in the Table-II. All of the strains were found to be sensitive to vancomycin, telavancin, dalbavancin, ceftobiprole, daptomycin, linezolid, quinuprustin/dalfopristin, trimetoprim/sulfametoxazole, rifampicin, and tigecycline. The susceptibility rate of the strains was found to be 92% for ceftaroline, 88% for minocycline, 84% for amikacin, 76% each for clindamycin and tobramycin and 48% for azithromycin.

**Table-II:** The MIC_50_, MIC_90_, MIC_interval_ (mg/L) values, sensitivity rates of 25 MRSA strains.

Agents	MIC_50_	MIC_90_	MIC_interval_	Sensitivity [n (%)]
VA*	0.75	1	0.5-1	25 (100)
TLV**	0.023	0.032	0.016-0.047	25 (100)
DAL	0.064	0.064	0.032-0.125	25 (100)
BPR*	0.75	1	0.38-1.5	25 (100)
CPT*	0.5	1	0.19-1.5	23 (92)
DAP	0.38	0.75	0.19-1	25 (100)
LNZ	1	1.5	0.38-1.5	25 (100)
AZM	24	>256	0.5->256	12 (48)
CD	0.19	>256	0.064->256	19 (76)
Q/D	1	1.5	0.5-1.5	25 (100)
SXT	0.032	0.38	0.012-1	25 (100)
RİF	0.012	0.016	0.006-0.094	25 (100)
MN	0.094	3	0.032-16	22 (88)
TGC	0.125	0.38	0.047-0.5	25 (100)
AK*	2	32	1-64	21 (84)
TOB*	0.5	>256	0.125->256	19 (76)

*: for S. aureus;

**: for MRSA.

**Fig.1(a-c):**
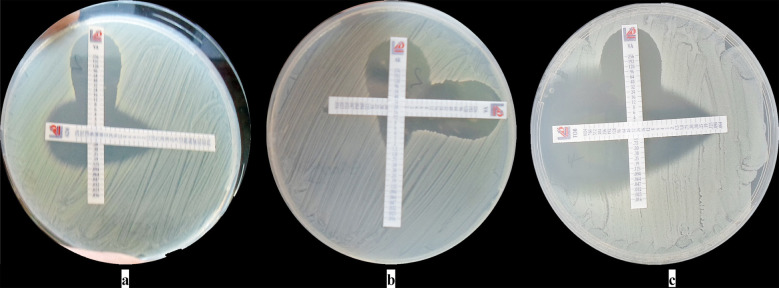
Some combination results from the study.

The interaction of vancomycin with other antimicrobials has been shown in the Table-III. Synergistic interaction was detected in five strains for VA-SXT, three strains for VA-AZM, in two each strains for VA-LNZ and VA-MN, and in one strain each for VA-DAL, VA-CD and VA-TOB combinations. The additive effect was detected in all MRSA strains (25) for VA-TLV; 24 strains for VA-DAL; 23 each for VA-CPD, VA-DAP, and VA-QDA; 22 each for VA-BPR and VA-AK; 20 each for; VA-LNZ, VA-MN, and VA-TOB; 19 each for VA-SXT, and VA-TGC, 18 each for VA-CD and VA-RİF; and 14 for VA-AZM. Indifference interaction was generally detected at low rates. Some of the results of the combination assays has been shown in the Fig.1 (a-c). No antagonistic interaction was detected in any of the combinations except against one strain for VA-AK.

**Table-III:** Interactions of antibiotics with vancomycin.

A	Synergy	Additive	Indifference	*Antagonism*

Combinations	n (%)	n (%)	n (%)	n (%)

VA-TLV^1^	0 (0)	25 (100)	0 (0)	0 (0)
VA-DAL^2^	1 (4)	24 (96)	0 (0)	0 (0)
VA-BPR^3^	0 (0)	22 (88)	3 (12)	0 (0)
VA-CPT^4^	0 (0)	23 (92)	2 (8)	0 (0)
VA-DAP^5^	0 (0)	23 (92)	2 (8)	0 (0)
VA-LNZ^6^	2 (8)	20 (80)	3 (12)	0 (0)
VA-AZM^7^	3 (12)	14 (56)	8 (32)	0 (0)
VA-CD^8^	1 (4)	18 (72)	6 (24)	0 (0)
VA-QDA^9^	0 (0)	23 (92)	2 (8)	0 (0)
VA-SXT^10^	5 (20)	19(76)	1 (4)	0 (0)
VA-RİF^11^	0 (0)	18 (72)	7 (28)	0 (0)
VA-MN^12^	2 (8)	20 (80)	3 (12)	0 0
VA-TGC^13^	0 (0)	19 (76)	6 (24)	0 (0)
VA-AK^14^	0 (0)	22 (88)	2 (8)	1 (4)
VA-TOB^15^	1 (4)	20 (80)	4 (16)	0 (0)

* 1:Vancomycin/telavancin, 2:Vancomycin/dalbavancin, 3: Vancomycin/ceftobiprole, 4:Vancomycin/ceftaroline, 5:Vancomycin/daptomycin, 6:Vancomycin/linezolid, 7:Vancomycin/ azithromycin, 8:Vancomycin/clindamycin, 9:Vancomycin/quinupristin&dalfopristin, 10:Vancomycin/trimethoprim-sulfamethoxazole, 11:Vancomycin/rifampicin, 12:Vancomycin/ minocycline, 13:Vancomycin/tigecycline, 14:Vancomycin/amikacin, 15:Vancomycin/tobramycin

## DISCUSSION

Combination of synergistic antimicrobials is frequently used in order to increase the spectrum an in the treatment. A number of methods that can be used to detect in-vitro synergy between antibiotics has been described. The E-test method has been evaluated as a good alternative for investigating the effect of the combination because of its ease of application, evaluation and good agreement with the standard checkerboard methodology.^10^,^14^,^16^

In the present study in which E-test methodology was used, the synergistic results obtained from the combinations were either at low rates or none. The synergistic effect was determined as 20% for VA-STX, 12% for VA-AZM, 8% each for VA-LNZ and for VA-MN, 4% each for VA-DAL, VA-CD and VA-TOB. Interaction results were obtained as additive at higher rates and indifference at lower rates. Parallel to the similar studies, the newer antimicrobials were found to have higher activity against MRSA strains than the convensional antimicrobials.^17^,^18^

The results of similar studies found in literature are also summarized below. In a study that investigated the effects of vancomycin and linezolid in combination using two different methods, VA-LNZ the antibiotics were reported to demonstrate indifference effect by checkerboard method, but synergistic effect by time-kill assays.^19^ In other studies that was performed using time-kill assay no synergy has been observed, but antagonism or indifference.^20^,^21^ In the present study, synergy, additive and indifferent interactions have been determined in 2, 20 and three strains, respectively. Similar to the results of the present study for VA-DAP combination, it has been reported that two antibiotics in combination have either indifference or additive effect, but not synergy.^22^ In another study, vancomycin and tigecycline combination has been tested against MRSA, and no difference in bactericidal activity was observed between combination compared with vancomycin or tigecycline alone.^23^ In present study, VA-TGC combination resulted in additive effect in 19 and indifference effect in six of 25 MRSA strains. Vancomycin and quinupristin/dalfopristin has been reported to reveal both antagonistic and synergistic effects.^24^ In the present study, 23 additives, two indifference effects and no antagonisitic effect were observed for the combination of two antibiotics. Ceftobiprole and ceftaroline are novel, broad-spectrum cephalosporins with in vitro activities against staphylococci, including MRSA. In the present study, MIC_50_ and MIC_90_ values have been recorded as 0.75 and 1mg/L for ceftobiprole and 0.50 and 1mg/L for ceftaroline, respectively. Additionaly, additive effect were detected for 22 and 23 of 25 MRSA strains and indifference effect was observed for three and two strains for the vancomycin combination of ceftobiprole and ceftaroline, respectively. No synergism and antogonism was detected. The results show that both antimicrobials have excellent activities against MRSA strains. The effect of vancomycin combination with trimetoprim-sulfamethoxazole and rifampicin against MRSA strains was evaluated by da Silva LV *et al*.^15^ Synergistic effect were recorded in three strains and none, respectively. In the present study, synergistic interaction of vacomycin with trimetoprim-sulfamethoxazole and rifampicin has been detected against five MRSA strains and none, respectively. Similar to the results of our study, Miranda-Novales *et al*.^25^ reported that vancomycin and amikacin generally revealed additive effect. On the other hand, antagonistic interaction was observed for one strain in the present study.

## CONCLUSION

The increase in the activity of classical and newer antibiotics studied in this study in combination with vancomycin is promising for the contributions in the treatment of infections caused by MRSA strains, taking into consideration that vancomycin has been used alone in the treatment of MRSA infections for many years, and as a result the resistance among the strains and the failure rate of the treatment has further increased over time. Additionaly, it is of importance to consider the advantage of the combination treatments to reduce and/or delay the development of resistance, it would be appropriate to carry out further studies and clarify the beneficial/supportive effects. The absence of antagonistic effect, except one strain for VA-AK combination, and generally additive / indifference results were evaluated as combinations used in the study would be useful in the treatment of MRSA infection.
